# Comparison of penetrating femtosecond laser-assisted astigmatic keratotomy and toric intraocular lens implantation for correction of astigmatism in cataract surgery

**DOI:** 10.1038/s41598-021-86763-5

**Published:** 2021-04-01

**Authors:** Hoon Noh, Young-Sik Yoo, Kyoung Yoon Shin, Dong Hui Lim, Tae-Young Chung

**Affiliations:** 1 Department of Ophthalmology, Samsung Medical Center, Sungkyunkwan University School of Medicine, 81 Irwon-Ro, Gangnam-gu, Seoul, 06351 Republic of Korea; 2 Department of Ophthalmology, Uijeongbu St. Mary’s Hospital, College of Medicine, The Catholic University of Korea, Uijeongbu-si, Gyeonggi-do Republic of Korea; 3 Department of Ophthalmology, Seongnam Citizens Medical Center, Seongnam-si, Gyeonggi-do Republic of Korea; 4 Department of Medical Device Management and Research, Samsung Advanced Institute for Health Sciences and Technology, Sungkyunkwan University, Seoul, Republic of Korea

**Keywords:** Corneal diseases, Lens diseases, Refractive errors

## Abstract

This study tried to compare the clinical outcomes of femtosecond laser-assisted astigmatic keratotomy (FSAK) and toric intraocular lens (IOL) implantation for astigmatism correction and identify factors affecting the efficacy of FSAK and toric IOL implantation in astigmatism correction. This retrospective case series comprised patients with corneal astigmatism ranging between 0.5 D and 4.5 D. Patients underwent FSAK or toric IOL implantation for cataract treatment and correction of astigmatism at the Samsung Medical Center, a tertiary surgical center, between April 2016 and December 2018. All patients underwent examination before and at three months after the surgery for comparative evaluation of refractive astigmatism, corneal high order aberrations and irregularity index. The astigmatism correction was analyzed by the Alpins method. Subgroup analysis of preoperative factors was based on the extent of target-induced astigmatism (TIA), the degree of astigmatism, and astigmatism classification based on topography. Thirty-one eyes underwent toric IOL implantation and 35 eyes underwent FSAK. The refractive astigmatism was significantly decreased in both toric IOL (*P* = 0.000) and FSAK group (*P* = 0.003). The correction index (CI) of refractive astigmatism was 0.84 ± 0.39 in the toric IOL and 0.71 ± 0.60 in the FSAK group. There was no difference between the two groups (*P* = 0.337). The CI of the FSAK group was significantly lower than in the toric IOL group when TIA was more than 1.5 D (*P* = 0.006), when correcting against-the-rule (*P* = 0.017), and limbus-to-limbus astigmatism (*P* = 0.008). In conclusion, toric IOL implantation is an effective and safe procedure for correcting preoperative astigmatism in cataract surgery in the short-term observation.

## Introduction

Advances in refractive cataract surgery have increased the demand for precision among both doctors and patients. Cataract surgery can be used simultaneously to correct refractive errors such as myopia, astigmatism and presbyopia, along with recovery of vision and improved quality of life. However, astigmatism remains an important obstacle to achieving emmetropia. One-third to one-half of all patients undergoing cataract surgery exhibit corneal astigmatism in need of correction^1–4^ and 15% to 56% of patients manifest more than 1.0 diopter (D) of astigmatism after cataract surgery^5^. Astigmatism, even at relatively low levels, can produce glare, monocular diplopia and visual distortions. Treating preoperative corneal astigmatism to meet patients’ needs for complete visual rehabilitation is an ophthalmic challenge.

Astigmatism after cataract surgery can usually be managed using a toric intraocular lens (IOL), and several studies have evaluated the success of toric IOL implantation^6–9^. In addition to toric IOL implantation, astigmatism can be corrected during and after cataract surgery, by either manual or femtosecond laser-assisted astigmatic keratotomy (FSAK), which is usually recommended for low-to-moderate astigmatism^10–13^. FSAK uses a femtosecond laser to make arcuate, paired or unpaired partial-thickness incisions on steep corneal meridian^14,15^. FSAK can be used to create incisions with accurate angle, depth and location, which greatly improves the predictability and accuracy of corneal astigmatism correction compared to conventional manual astigmatic keratotomy (AK)^16,17^.

When correcting astigmatism using AK, the patient's age, the degree of astigmatism, the type of incision (penetrating or intrastromal), and corresponding nomogram are considered. Several studies have compared the effects of toric IOL implantation and FSAK astigmatism correction, but the factors underlying the astigmatism correction by these methods have yet to be elucidated.

Therefore, we compared the clinical outcomes of FSAK to those of toric IOL implantation for correcting astigmatism in cataract surgery and to identify factors affecting the efficacy of FSAK and toric IOL implantation in correcting astigmatism.

## Methods

This study was approved by the Institutional Review Board (IRB)/Ethics Committee of Samsung Medical Center (reference 2016-11-095) and adhered to the tenets of the Declaration of Helsinki. The IRB of Samsung Medical Center approved the waiver of informed consent in this study. This retrospective case series included 66 eyes of 66 patients who underwent cataract surgery between April 2016 and December 2018 at the cataract and refractive clinic of Samsung Medical Center.

All 66 patients had visually significant cataract and regular corneal astigmatism values measured with Scheimpflug imaging (Pentacam HR, Oculus) between 0.5 D and 4.5 D, including both anterior and posterior corneal surface using vector summation^18^. None of the patients had any ocular or systemic contraindications to surgery. Exclusion criteria were amblyopia, irregular astigmatism, corneal opacity, glaucoma, retinal disease, history of ocular inflammation, history of ocular trauma and past exposure to other intraocular surgeries. The patients were divided into two groups, the FSAK and toric IOL group, and their clinical data and astigmatism correction parameters were compared.

### Preoperative and postoperative examinations

All patients underwent examinations before surgery and 3 months after surgery, performed by the same ophthalmic technician. Preoperatively, all patients underwent extensive ophthalmic evaluation that included slit-lamp examination, tonometry, corrected distance visual acuity (CDVA), manifest refraction, dilated fundoscopy, non-contact specular microscopy (Non-Con Robo SP 6000, Konan Medical Inc.), corneal topography and aberrometry using Scheimpflug imaging (Pentacam HR, software version 1.22r05, Oculus). Corneal irregularity index (IR) was automatically calculated in µm scale via a Fourier analysis map, whereas high-order aberrations (HOAs) in the 6 mm zone were automatically calculated in µm scale using the Cataract map of Pentacam. The corneal astigmatism was divided into central and limbus-to-limbus astigmatism. The limbus-to-limbus astigmatism is the case where the typical bow-tie shape was extended to the limbus in the Pentacam examination and defined as the state where the corneal astigmatism was extended beyond the central 6 mm zone to the periphery. Otherwise, it was defined as central astigmatism. The same ophthalmic examinations were repeated at 3-month follow up. Preoperative and postoperative refractive and corneal astigmatism were calculated, and a vector analysis of the astigmatic changes was performed using the Alpins’ vector method^19,20^.

Biometry measurements (axial length and anterior chamber depth) used for IOL power calculation were obtained using optical coherence biometry (IOLMaster 700, software version 1.70, Carl Zeiss Meditec AG). Total corneal astigmatism was calculated based on both anterior and posterior corneal surface measurements via Scheimpflug imaging (Pentacam HR, software version 1.22r05, Oculus) using vector summation according to the Alpins’ method^18–21^. Based on these data, the cylindrical power and axis placement in the toric IOL group were calculated using the online tools developed by the IOL manufacturer. The calculated corneal astigmatism was used to determine the AK profile of the FSAK group.

### Surgical technique

#### Toric IOL implantation

Before surgery, a 0°–180° axis was marked with all patients seated upright in front of slit-lamp using a horizontal slit beam. Intraoperatively, the intended implantation axis was marked on the limbus after correctly aligning a Mendez ring with the primary marks to ascertain the intended angle of placement according to preoperative plan. A single experienced surgeon (T.Y.C.) performed all surgeries under topical anesthesia with Alcaine (proparacaine hydrochloride ophthalmic solution) 0.5%. Phacoemulsification was performed through a 2.75 mm temporal clear corneal incision. After performing continuous curvilinear capsulorhexis with an intended diameter of 5.0 mm and hydrodissection, phacoemulsification of the nucleus and bimanual aspiration of the residual cortex were performed using a cataract surgery phacoemulsification device (Centurion Vision System, Alcon). Toric IOL (IQ toric IOL, Alcon) was implanted in the capsular bag using an injector and disposable cartridge system before removing the ophthalmic viscosurgical device (OVD). After removing the OVD, the IOL was rotated to its final targeted position by perfectly aligning the toric reference marks on the IOL surface with the limbal axis marks. The Alcon online toric IOL calculator (available from: http://www.myalcon-toriccalc.com) was used with A-constant of 119.0 based on the calculated total corneal astigmatism. Finally, a balanced salt solution was injected into the incision site to close the corneal incision, causing edema. After the surgery, postoperative eye drops of antibiotics (gatifloxacin 0.3%, Gatiflo; Handok) and corticosteroid (lotepredrol etabonate, Lotemax; Bausch + Lomb) were used 4 times daily and tapered over a month^18^.

#### Femtosecond laser-assisted cataract surgery (FLACS) combined with FSAK

The Alcon Verion Image Guided System was used to capture the preoperative anatomic landmarks of the eye on the day of surgery. All surgeries were performed by the same experienced surgeon (T.Y.C.) under topical anesthesia (proparacaine hydrochloride 0.5%, Alcaine; Alcon) with the femtosecond laser platform (LenSx, Alcon) and the phacoemulsification device. The patients were placed in a supine position and a speculum was placed to open the eye. Docking and suction procedures were completed by adjusting the position of the patient interface (SoftFit, Alcon) to ensure that the curved contact lens applanated the cornea. A spectral-domain optical coherence tomography (OCT) imaging device was utilized to scan the patient’s eye and locate the specific target areas. Laser treatment was performed after manual verification of each procedural step (corneal incisions, capsulotomy, and lens fragmentation). The patient was then transferred for the subsequent operation. After the main corneal incision was separated surgically using spatulas, the anterior chamber was filled with a viscoelastic solution. Next, the cut anterior capsule was removed using a capsulorhexis forcep. Hydrodissection was performed followed by phacoemulsification of the nucleus and aspiration of the residual cortex using the phacoemulsification device. Finally, a monofocal aspheric foldable IOL (Acrysof IQ; Alcon) was implanted in the capsular bag and the corneal incisions were hydrated^22^.

#### FSAK design

**Figure 1:**
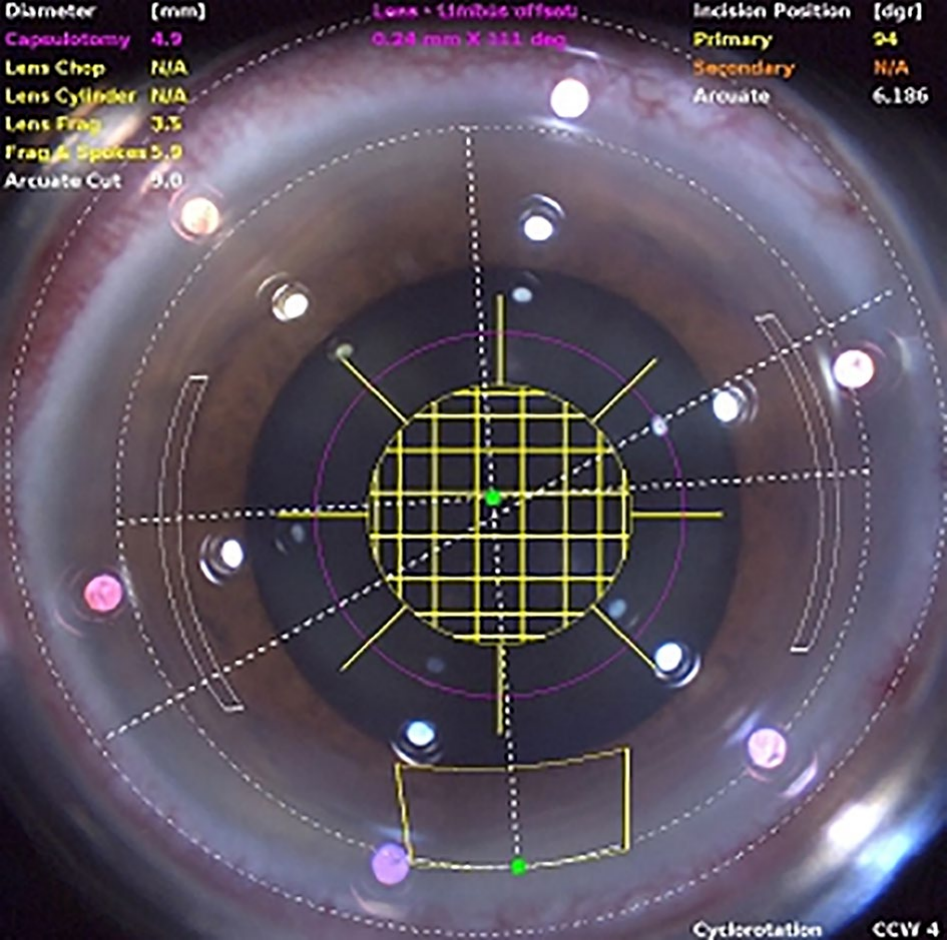
An example of programmed femtosecond laser-assisted astigmatic keratotomy in femtosecond laser assisted cataract surgery.

Phacoemulsification combined with AK was performed using the femtosecond laser platform guided by real-time intraoperative spectral-domain OCT. Based on the measurements of corneal astigmatic axis using preoperative corneal topography, the arcuate incision was made with the femtosecond laser. All treatments were paired with symmetric incisions centered on the steep axis. The width of FSAK was calculated using a modified nomogram increased by 30% from the nomogram of the femtosecond laser system. The penetrating keratotomy incision was made at a corneal thickness depth of 85% and an arc diameter of 9.0 mm. The primary corneal incision showed a tri-planar configuration with a width of 2.75 mm and was located at the superior or temporal corneal meridian. An example of the programmed FSAK in FLACS is shown in Fig. 1. The AK incisions were not opened.

### Analysis of astigmatic correction

The magnitude and axis of keratometric astigmatism were calculated according to both anterior and posterior corneal surface measured with a Pentacam HR using vector summation to determine the cylindrical power and axis location of the toric IOL group and the AK profiles of the FSAK group. The magnitude and axis of refractive astigmatism were used to analyze the postoperative outcome of astigmatism correction. The astigmatic analyses were performed using the Alpins method^19,20^. In this study, the target-induced astigmatism (TIA) was defined as the intended astigmatic correction with the magnitude and angle. TIA was calculated using a toric IOL calculator in the toric IOL group or a nomogram calculator in the FSAK group incorporating the value of keratometric astigmatism and the surgeon-specific flattening effect (− 0.5 D) of 2.75 mm main incision. The surgically-induced astigmatism (SIA) represents the refractive astigmatic correction converted to the corneal plane achieved by the toric IOL or AK. The difference vector (DV) is the induced astigmatic change that facilitates the intended target acquisition in the initial surgery. The DV is an absolute measure of success and is preferably zero. Furthermore, the relationships between the three fundamental vectors were calculated at follow-up: correction index (CI), the ratio of SIA to TIA (CI > 1 indicates overcorrection; CI < 1 indicates undercorrection).

### Statistical analysis

A statistical analysis program (SPSS version 24, SPSS Inc.) was used to analyze the data. Independent *t*-tests and Pearson’s χ^2^ tests were used to compare preoperative patient demographics and baseline values between the toric IOL and FSAK groups. The preoperative and postoperative clinical data, astigmatism correction parameters, corneal irregularity parameters, and corneal ECD were compared between the toric IOL and FSAK groups, using independent *t*-tests. A *p*-value < 0.05 was considered significant.

### Ethical approval

All procedures performed in studies involving human participants were in accordance with the ethical standards of the IRB of Samsung Medical Center and complied with the 1964 Helsinki declaration and its later amendments or comparable ethical standards.

## Results

This retrospective case series identified 66 patients (66 eyes) including 34 men and 32 women between ages 23 and 87 years). Four patients (4 eyes) in the toric IOL group and 6 patients (6 eyes) in the FSAK group were younger than 45 years of age.

Thirty-one patients (31 eyes) and 35 patients (35 eyes) underwent toric IOL implantation and FLACS and AK. There were no significant differences between the toric IOL group and FSAK group in patients’ age, endothelial cell density, spherical equivalent (SE), refractive astigmatism or corneal astigmatism (Table 1). The value of maximum corneal astigmatism was 3.33 D in the toric IOL group and 4.02 D in the FSAK group.

**Table 1:** Preoperative patient demographics and baseline values.

	Toric IOL (N = 31)	FSAK (N = 35)	*P*
**Mean age (years)**	65.47 ± 15.91	61.54 ± 15.97	0.135
**Female sex (N, %)**	14 (45.2%)	18 (51.4%)	0.662
**Right eyes (N, %)**	13 (41.9%)	13 (37.1%)	0.622
**SE refractive error (D)**			
Arithmetic mean	− 1.92 ± 3.26	− 2.90 ± 3.53	0.232
Absolute mean	2.56 ± 2.77	3.32 ± 3.14	0.264
**Cylindrical refractive error (D)**	− 1.57 ± 1.19	− 1.52 ± 1.18	0.845
**Axial length (mm)**	24.34 ± 1.57	24.91 ± 2.32	0.163
**Mean corneal keratometry (D)**	44.0 ± 1.73	42.93 ± 7.75	0.428
**Mean corneal astigmatism (D)**	1.70 ± 0.78	1.52 ± 0.67	0.280
**Central corneal ECD (cells/mm**^**2**^**)**	2870.3 ± 290.6	2830.7 ± 337.3	0.613

*MAR* minimal angle of resolution, *D* diopter, *RMS* root-mean square, *ECD* endothelial cell density.

**Figure 2:**
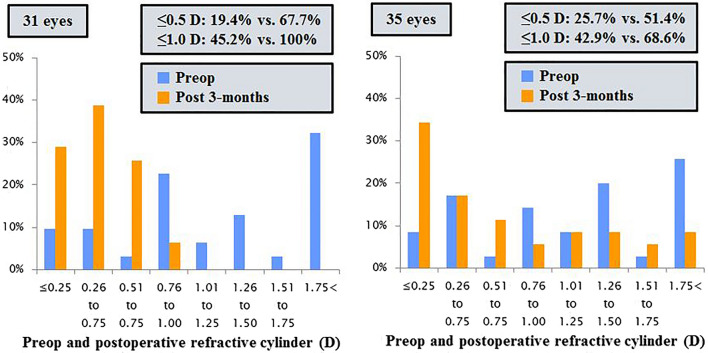
Preoperative and postoperative refractive astigmatism in the toric IOL group (*left*) and FSAK group (*right*) (*IOL* intraocular lens, *FSAK* femtosecond laser-assisted astigmatic keratotomy).

A significant difference was found between preoperative and postoperative refractive astigmatism in the toric IOL and FSAK groups (*P* = 0.000, *P* = 0.003, respectively). Figure 2 presents the refractive outcomes graphically. Twenty-one patients with toric IOL (67.7%) and 18 FSAK patients (51.4%) attained a postoperative refractive cylinder of less than 0.5 D and 31 patients (100%) compared with 24 patients (68.6%) had less than 1.0 D. The toric IOL group exhibited a significantly lower postoperative refractive cylinder (0.46 ± 0.32 D vs. 0.80 ± 0.73 D; *P* = 0.017) compared with the FSAK group. There was a postoperative increase in root mean square (RMS) HOAs and IR in the FSAK group (*P* = 0.016 and *P* = 0.000), but no significant changes in the toric IOL group (*P* = 0.903 and *P* = 0.754). Moreover, the degree of changes before and after surgery increased in the FSAK group (0.133 ± 0.251 µm) but decreased in the toric IOL group (− 0.016 ± 0.276 µm) at 3 months, which showed significant difference (*P* = 0.020). The postoperative central corneal endothelial cell density (ECD) did not significantly differ between the 2 groups (Table 2).

**Table 2:** Postoperative changes in astigmatism and corneal aberrations.

	Toric IOL (N = 31)	*P*^a^	FSAK (N = 35)	*P*^a^	*P*^b^
**Cylindrical refractive error (D)**					
Preop	− 1.57 ± 1.19		− 1.52 ± 1.18		0.845
Postop	− 0.46 ± 0.32		− 0.80 ± 0.73		0.017*
Postop vs. preop		0.000*		0.003*	
**Vector analysis**					
TIA (D)	1.70 ± 0.78		1.52 ± 0.67		0.323
SIA (D)	1.45 ± 1.10		1.00 ± 0.77		0.075
DV (D)	1.08 ± 0.69		1.49 ± 0.84		0.026*
CI	0.84 ± 0.39		0.71 ± 0.60		0.337
**Central corneal ECD (cells/mm**^**2**^**)**	2,612.1 ± 351.4		2,607.4 ± 338.4		0.529
**High-order aberrations, RMS (µm)**					
Preop	0.842 ± 0.562		0.577 ± 0.238		0.019*
Postop	0.826 ± 0.457		0.709 ± 0.211		0.197
Postop vs. preop		0.903			
Δ^c^	− 0.016 ± 0.276		0.133 ± 0.251	0.016*	0.020*
**Irregularity index (µm)**					
Preop	0.040 ± 0.025		0.035 ± 0.014		0.268
Postop	0.042 ± 0.022		0.052 ± 0.014		0.043*
Postop vs. preop		0.754		0.000*	

*TIA* target-induced astigmatism, *D* diopter, *SIA* surgically induced astigmatism, *DV* difference vector, *CI* correction index, *ECD* endothelial cell density, *RMS* root-mean square.

^a^Comparison with baseline, P < 0.05 was considered statistically significant.

^b^Comparison of both groups at each time.

^c^Postop–preop.

**Figure 3:**
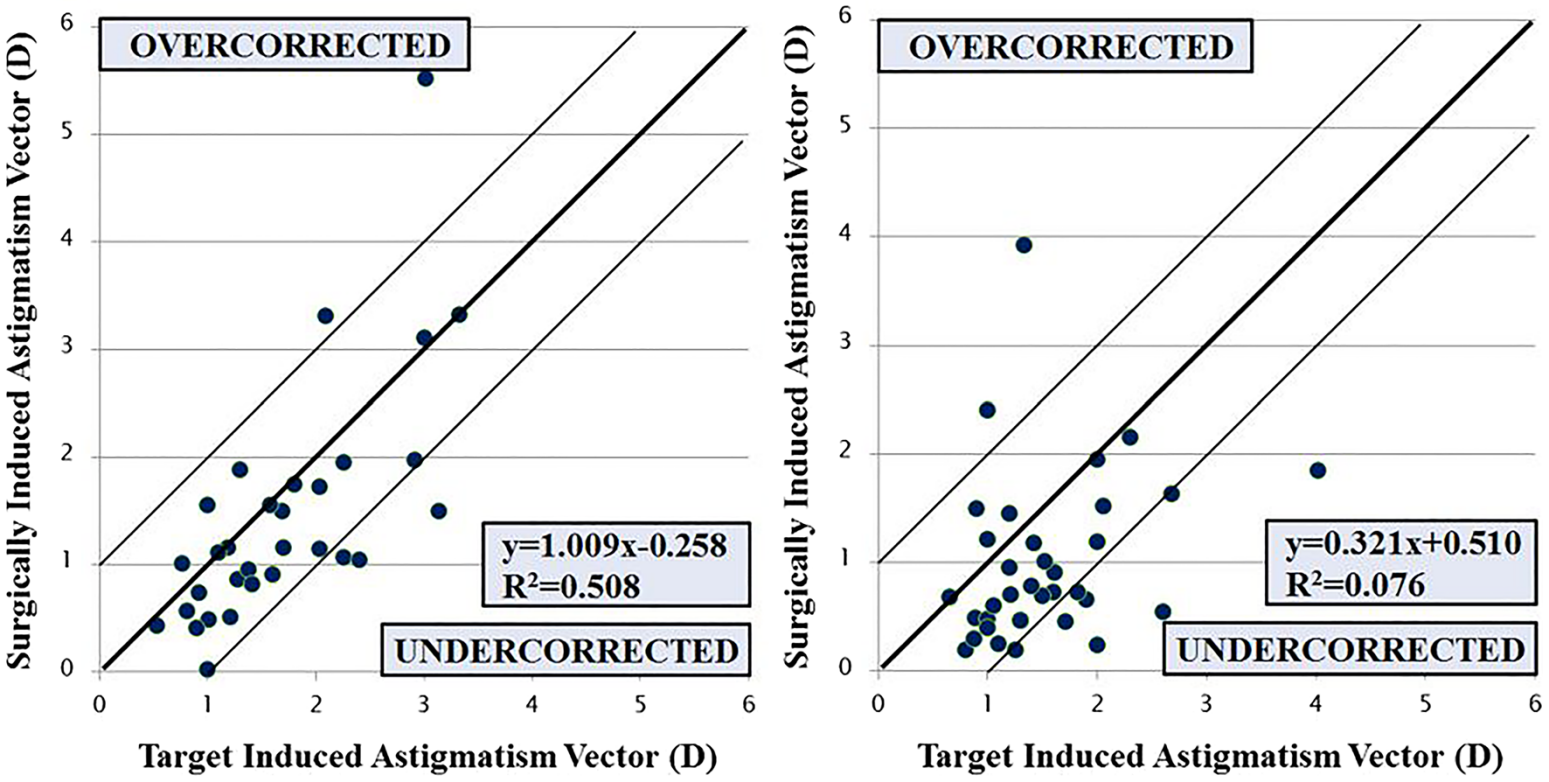
TIA versus SIA plots (refractive astigmatism) of patients treated with toric IOL implantation (*left*) and femtosecond laser-assisted astigmatic keratotomy (*right*). The thin lines represent the range within ± 1.0 D, respectively, between 2 parameters. The rate of strong overcorrection (above the superior thin line) and undercorrection (below the inferior thin line) was not statistically different between the two groups (*TIA* target-induced astigmatism; *SIA* surgically induced astigmatism).

Vector analysis of refractive astigmatism showed a mean TIA of 1.70 ± 0.78 D and a mean SIA of 1.45 ± 1.10 D in the toric IOL group. In the FSAK group, the mean TIA was 1.52 ± 0.67 D and the mean SIA was 1.00 ± 0.77 D. The SIA was less than TIA in both groups, indicating undercorrection. There was no difference in the correction index (CI) between the 2 groups. The DV was lower in the toric IOL group (*P* = 0.036) indicating better correction (Table 2). A scatter plot of TIA versus SIA at 3 months after toric IOL implantation and FLACS combined with FSAK is presented in Fig. 3, which shows undercorrection. Three (9.7%) of the 31 eyes in the toric IOL group and 8 (22.9%) of 35 eyes in the FSAK group were strongly undercorrected (CI close to 0.5, below the inferior thin line in Fig. 3) at 3 months (*P* = 0.152). The difference between the magnitudes of TIA and SIA was less than 1.0 D in 26 (83.9%) of 31 eyes in the toric IOL group and in 25 (71.4%) of 35 eyes in the FSAK group (*P* = 0.383).

The subgroup analysis of postoperative changes in astigmatism based on the magnitude of TIA reveals a significantly decreased CI of the FSAK group compared with the toric IOL group when TIA was greater than 1.5 D (*P* = 0.006). The mean postoperative refractive cylinder was significantly higher in the FSAK group (1.03 ± 0.80 D) than in the toric IOL group (0.41 ± 0.38 D) at 3 months (*P* = 0.014). When the TIA was less than 1.5 D, the mean postoperative refractive cylinder, SIA, and CI showed no statistically significant differences between groups (Table 3). In addition, the corneal astigmatism was divided into two forms, with-the-rule (a steep axis within 30° of the vertical meridian, WTR) and against-the-rule (a steep axis within 30° of the horizontal meridian, ATR). The respective astigmatism correction parameters were compared. The CI of the FSAK group was significantly lower than that of the toric IOL group when correcting ATR astigmatism (*P* = 0.017). The mean postoperative refractive cylinder was significantly higher in the FSAK group (1.00 ± 0.55 D) than in the toric IOL group (0.53 ± 0.32 D) at 3 months (*P* = 0.035). The mean postoperative refractive cylinder, SIA and CI showed no statistically significant differences between groups when correcting for WTR astigmatism (Table 3). Finally, the corneal astigmatism was divided into central and limbus-to-limbus astigmatism, and the respective astigmatism correction parameters were compared. The CI of the FSAK group was significantly lower than that of the toric IOL group when correcting for limbus-to-limbus astigmatism. The mean postoperative refractive cylinder was significantly higher in the FSAK group (0.95 ± 0.83 D) than in the toric IOL group (0.48 ± 0.31 D) at 3 months (*P* = 0.030). The mean postoperative refractive cylinder, SIA and CI showed no statistically significant differences between groups when correcting for central astigmatism (Table 3).

**Table 3:** Subgroup analysis of postoperative changes in refractive astigmatism.

	Toric IOL	FSAK	*P*	Toric IOL	FSAK	*P*
	TIA < 1.5 D			1.5 D ≤ TIA		
**Number of eyes**	15	20		16	15	
**Cylindrical refractive error (D)**						
Preop	0.88 ± 0.59	1.25 ± 1.15	0.314	2.22 ± 1.23	1.88 ± 1.16	0.446
Postop	0.52 ± 0.24	0.63 ± 0.65	0.633	0.41 ± 0.38	1.03 ± 0.80	0.014*
**Vector analysis**						
TIA (D)	1.05 ± 0.25	1.09 ± 0.21	0.601	2.30 ± 0.60	2.01 ± 0.64	0.202
SIA (D)	0.83 ± 0.47	0.93 ± 0.89	0.587	2.04 ± 1.22	1.09 ± 0.60	0.008*
DV (D)	0.93 ± 0.51	1.43 ± 1.02	0.131	1.23 ± 0.82	1.57 ± 0.52	0.177
CI	0.80 ± 0.41	0.85 ± 0.74	0.419	0.87 ± 0.39	0.52 ± 0.25	0.006*

	WTR astigmatism			ATR astigmatism		
**Number of eyes**	20	25		9	10	
**Cylindrical refractive error (D)**						
Preop	1.44 ± 1.31	1.41 ± 1.28	0.926	1.94 ± 0.96	1.80 ± 0.90	0.842
Postop	0.45 ± 0.32	0.72 ± 0.79	0.726	0.53 ± 0.32	1.00 ± 0.55	0.035*
**Vector analysis**						
TIA (D)	1.77 ± 0.91	1.48 ± 0.69	0.424	1.58 ± 0.46	1.62 ± 0.62	0.905
SIA (D)	1.50 ± 1.25	1.05 ± 0.87	0.112	1.48 ± 0.77	0.88 ± 0.45	0.043*
DV (D)	1.18 ± 0.70	1.63 ± 0.90	0.071	0.71 ± 0.44	1.13 ± 0.55	0.079
CI	0.83 ± 0.39	0.76 ± 0.69	0.061	0.94 ± 0.37	0.58 ± 0.23	0.017*

	**Central astigmatism**			**Limbus-to-limbus astigmatism**		
**Number of eyes**	16	15		15	20	
**Cylindrical refractive error (D)**						
Preop	1.14 ± 0.70	1.05 ± 0.56	0.695	2.03 ± 1.45	1.88 ± 1.40	0.746
Postop	0.44 ± 0.34	0.60 ± 0.53	0.470	0.48 ± 0.31	0.95 ± 0.83	0.030*
**Vector analysis**						
TIA (D)	1.41 ± 0.56	1.21 ± 0.36	0.258	2.01 ± 0.88	1.75 ± 0.75	0.364
SIA (D)	1.00 ± 0.56	0.83 ± 0.57	0.399	1.94 ± 1.34	1.13 ± 0.89	0.025*
DV (D)	1.13 ± 0.71	1.36 ± 0.81	0.417	1.03 ± 0.69	1.60 ± 0.87	0.047*
CI	0.71 ± 0.32	0.75 ± 0.61	0.446	0.97 ± 0.43	0.67 ± 0.60	0.008*

*TIA* target-induced astigmatism, *D* diopter, *SIA* surgically induced astigmatism, *DV* difference vector, *CI* correction index, *WTR* within-the-rule, *ATR* against-the-rule.

Femtosecond laser treatment was delivered successfully in all cases. None of the AKs resulted in complications, including posterior perforation or inadvertent placement. No IOL was misaligned more than 10° in the toric IOL group (2.03 ± 0.76°).

## Discussion

Our study showed a significant reduction in refractive astigmatism after surgery in both toric IOL and FSAK groups. The FSAK group showed similar results in postoperative cylindrical refraction error and CI during the correction of low-to-moderate, within-the-rule or central corneal astigmatism compared with the toric IOL group. The FSAK group showed no differences in corneal endothelial cell density before and after laser ablation.

The mean astigmatism correction was 84% and 71%, with 6.4% and 5.7% of eyes exhibiting extended overcorrection (CI close to 2.0, above the superior thin line in Fig. 3) in the toric IOL and FSAK groups, respectively, which was in line with other similar studies, demonstrating the effectiveness of FSAK for correcting astigmatism in cataract surgery. Yoo et al.^23^ reported that the refractive astigmatism decreased significantly from 1.71 D to 0.78 D when penetrating FSAK was applied to the cornea (diameter = 9.0 mm; depth = 85%) to correct residual astigmatism after cataract surgery using a 60-kHz IntraLase femtosecond laser. The investigators compared with toric IOL implantation in cataract patients diagnosed with corneal astigmatism and like our study, found no significant difference in residual refractive astigmatism between the two treatment methods. Rückl et al.^24^ reported similar results, including a significant decrease in refractive astigmatism after FSAK, from 1.41 D to 0.33 D. FSAK was designed for paired arcuate cuts on steep axes completely within the corneal stroma, with a 7.5-mm arc diameter. Day et al.^12^ performed intrastromal FSAK in 196 eyes and reported a decrease in corneal astigmatism by 39% from 1.21 D preoperatively to 0.74 D postoperatively.

Several studies have also reported risk factors such as astigmatic undercorrection or overcorrection. In our study, the FSAK group tended to be undercorrected when compared with the toric IOL group, but there was no statistically significant difference (Fig. 3). In addition, the FSAK group had a significantly higher postoperative refractive cylinder compared with the toric IOL group (Table 2). Chang^25^ reviewed several related articles and evaluated the efficacy, complications, and various methods of FSAK used for astigmatism correction and concluded that it tends to be undercorrected when TIA is more than 1 D. In our study, TIA was less than 1 D in only 5 (14.3%) of 35 eyes in the FSAK group, which might be attributed to undercorrection. In addition, the effect of FSAK was significantly lower than that of toric IOL implantation when correcting moderate-to-high astigmatism (TIA ≥ 1.5 D), which was in line with previous studies (Table 3)^10–13^. Wang et al.^26^ reported that the overcorrection rate of penetrating FSAK was 14.9% at 3 months after surgery. Two-thirds of 14.9% of all overcorrected eyes showed preoperative WTR corneal astigmatism. The authors estimated that this overcorrection may be the result of ignoring the effects of posterior corneal astigmatism. A new nomogram was developed to account for the posterior corneal effect^26^, and the overcorrection was reduced to 6.7%. However, in our study, only 5.7% of eyes in the FSAK group were strongly overcorrected. Because the posterior astigmatism was considered in determining the amount of FSAK in our study, the overcorrection rate was smaller than that of Wang et al.^26^ When performing cataract refractive surgery and FSAK concurrently, the effect of the posterior cornea on total corneal astigmatism must be considered.

IR is a number or index representing the degree of irregularity in the corneal surface morphology. It is a measure of the standard deviation between the corneal surface and the best-fit reference surface. It can often be used to predict irregular astigmatism or visual distortions^27,28^. High values of this ratio indicate a greater possibility of corneal pathology related to morphological abnormality such as keratoconus. Evaluation of corneal aberration revealed a significant increase in RMS HOAs and IR in the FSAK group compared with the conventional toric IOL group. The aberration in our study was consistent with previous reports^29–32^. In our study, the FSAK might induce visual defects such as glare and halo by increasing HOAs and corneal irregularity compared with toric IOL implantation; however, the authors did not investigate the postoperative visual challenges. A further study is needed to elucidate the effect of FSAK on the quality of subjective vision.

Based on the subgroup analysis according to the direction of preoperative corneal astigmatism, the effect of FSAK was significantly lower than that of toric IOL implantation when correcting ATR astigmatism. When correcting WTR astigmatism, the primary corneal incision was located at the temporal corneal meridian and the AKs were paired with symmetric incisions centered on the vertical axis. However, when correcting ATR astigmatism, the main incision was located at the vertical meridian and the AKs were paired on the horizontal axis. In this case, regardless of the type of astigmatism, the primary corneal incision offsets the effect of astigmatism correction of AKs. It has been known for many years that a superior clear corneal incision induces substantially higher degree of astigmatism than temporal incision^33–36^. In our study, the effect of canceling astigmatism correction was greater in ATR astigmatism in which the main incision is vertical compared with the horizontal incision in WTR astigmatism, which was in line with the previous studies.

Traditionally, astigmatism is divided into roughly two types: central and limbus-to-limbus corneal astigmatism^37^. The limbus-to-limbus astigmatism is characterized by a typical bowtie shape extending to the limbus. No difference in astigmatism correction was found between the toric IOL and FSAK groups in central astigmatism, whereas in limbus-to-limbus astigmatism, the CI of FSAK group was significantly lower than in the toric IOL group. The viscoelastic properties of the cornea appear to play an important role in these results. The layered orientation of the human cornea has been associated with mechanical properties^38,39^. The mechanical effect increases in the direction of the meridian as it approaches the center of the cornea^39^. The pressure-induced meridian strain was the smallest at the corneal periphery^40^. Therefore, in limbus-to-limbus astigmatism, which requires corneal modification in the periphery, the effect of AK is relatively poor. In terms of orthokeratology (Ortho-K), spherical Ortho-K lenses have limited ability in rectifying limbus-to-limbus astigmatism. Ortho-K has a greater effect in reducing astigmatism in the central cornea compared with the peripheral cornea, which suggests that limbus-to-limbus astigmatism is difficult to treat compared to central astigmatism^41,42^, and is also explained by the viscoelastic properties of the cornea.

The femtosecond laser energy, which is irradiated close to endothelial cells, may affect the survival of endothelial cells. However, Rückl et al.^24^ and Hoffart et al.^43^ reported no significant endothelial cell loss after FSAK. Other reports suggested that the femtosecond laser reduced corneal endothelial cell damage and inflammatory response by decreasing the effective ultrasound time during phacoemulsification and thereby resulted in a favorable prognosis after surgery^44–47^. In our study, the postoperative central corneal ECD did not differ significantly between the toric IOL and FSAK groups (Table 2). In addition, when performing FSAK, we also recorded and compared the peripheral images of the corneal endothelium in four different quadrants (Table 4). No significant decrease was found in corneal ECD in the quadrant with primary incision or FSAK compared with the quadrant without, suggesting that FSAK was a safe procedure.

**Table 4:** Postoperative corneal endothelial cell density of the FSAK group.

	WTR astigmatism (vertically paired) (N = 25)	ATR astigmatism (horizontally paired) (N = 10)	*P*
**Corneal ECD (cells/mm**^**2**^**)**			
Central	2326.6 ± 248.5	2401.8 ± 295.4	0.752
Superior^a^	2112.9 ± 257.4	2048.3 ± 257.6	0.652
Inferior^b^	2191.5 ± 340.1	2393.7 ± 429.2	0.200
Temporal^c^	2081.6 ± 264.3	2281.3 ± 354.8	0.689
Nasal^d^	2332.9 ± 429.3	2191.8 ± 495.4	0.234

*WTR* within-the-rule, *ATR* against-the-rule, *ECD* endothelial cell density.

^a^The quadrant in which FSAK was done in the WTR astigmatism group and primary incision was done in the ATR astigmatism group.

^b^The quadrant in which FSAK was done in the WTR astigmatism group and no incision was done in the ATR astigmatism group.

^c^The quadrant in which primary incision was done in the WTR astigmatism group and FSAK was done in the ATR astigmatism group.

^d^The quadrant in which no incision was done in the WTR astigmatism group and FSAK was done in the ATR astigmatism group.

There are two important types of incision in FSAK. First, the penetrating FSAK performed in our study involves cutting from the anterior surface of cornea. The wound can be fully opened if the effect of the astigmatic correction was insufficient. Intrastromal FSAK is performed where the cut is within the stroma and does not reach the Bowman’s layer and epithelium. Intrastromal FSAK is associated with a minimal risk of infection, epithelial ingrowth or wound gape. In our study with penetrating FSAK, none of these complications were observed during the 3-month observation period, but a long-term study is needed. There is insufficient evidence suggesting that penetrating FSAK has a significantly greater effect than intrastromal FSAK due to differences in incision depth, incision arc length, and optical zone diameter for each relevant study, and the limited number of studies and data available^25^. In addition to our study, only one retrospective analysis to date compared penetrating FSAK and toric IOL implantation^23^. No study compared intrastromal FSAK and toric IOL implantation. Large scale randomized controlled trials with extended study periods are needed.

In conclusion, FSAK may be effective, predictable, and safe, and comparable to toric IOL implantation for correcting preoperative refractive astigmatism in cataract surgery in the short-term observation, but further long-term observation is needed. FSAK is a possible alternative to toric IOL implantation in patients with mild-to-moderate, WTR or central corneal astigmatism. However, toric IOL implantation is a more effective option than FSAK for moderate-to-high, ATR or limbus-to-limbus astigmatism.
